# Editorial

**Published:** 1854-07

**Authors:** 


### THE MEDICAL EXAMINER.

### PHILADELPHIA, JULY, 1854.

A full report of the proceedings of our State Medical Society, recently held at Pottsville, will be found in the present number. Sixteen Societies were represented, 59 delegates registering their names. A number of valuable reports were presented and read, and certain resolutions were passed, which will, if observed, greatly increase the value of its medical proceedings. We allude to the recommendations to County Societies on the proper method of preparing their reports, &c.

For the hospitality and kindness extended to us by the resident physicians, we tender our warmest thanks, and are sure that in so doing we but express the feeling of all our medical brethren who were present. The pure air and beautiful scenery of Pottsville, in the "leafy month" in which we visited it, presented to the Philadelphia delegation a most enviable and refreshing contrast to the confined atmosphere and crowded marts of their city.

The meeting lasted two days, and we are happy to state was in all respects an harmonious and satisfactory one.

